# Supplementary material for: Mechanistic insights into a TIMP3-sensitive pathway constitutively engaged in the regulation of cerebral hemodynamics
Source: eLife. 2016 Aug 1;5:e17536. doi: 10.7554/eLife.17536 (PMC4993587; doi:10.7554/eLife.17536)
Supplement: Figure 1—source data 1. — DOI: http://dx.doi.org/10.7554/eLife.17536.004 [file elife-17536-fig1-data1.docx]

## Figure 1- source data 1: Reagents used for Figure 1

| **Drug**  **(molecular weight, kDa)** | **Selectivity** | **Final concentration**  **(duration of superfusion)** |
| --- | --- | --- |
| **Murine TIMP1**  **(32kDa)** | Inhibits all MMPs, ADAM10 | 50 nM  (30 min) |
| **Murine TIMP2**  **(23 kDa)** | Inhibits all MMPs, ADAM12 | 50 nM  (30 min) |
| **Murine TIMP3**  **(24-28 kDa)** | Inhibits all MMPs, ADAM10, ADAM17 and ADAMTS | 8-40 nM  (30 min) |
