# Supplementary material for: Mechanistic insights into a TIMP3-sensitive pathway constitutively engaged in the regulation of cerebral hemodynamics
Source: eLife. 2016 Aug 1;5:e17536. doi: 10.7554/eLife.17536 (PMC4993587; doi:10.7554/eLife.17536)
Supplement: Figure 1—source data 2. — DOI: http://dx.doi.org/10.7554/eLife.17536.005 [file elife-17536-fig1-data2.docx]

**Figure 1- source data 2: Main physiological variables of mice studied in Figure 1**

| Genotype | Treatment  (concentration) | N | MAP  (mmHg) | pCO_2_  (mmHg) | pO_2_  (mmHg) | pH |
| --- | --- | --- | --- | --- | --- | --- |
| WT | Vehicle | 5 | 76±4 | 36±2 | 128±4 | 7.35±0.02 |
|  | TIMP3 (8 nM) | 5 | 75±4 | 35±2 | 122±5 | 7.34±0.02 |
| WT | Vehicle | 5 | 78±3 | 35±3 | 131±3 | 7.34±0.03 |
|  | TIMP3 (40 nM) | 5 | 75±5 | 34±2 | 123±5 | 7.33±0.02 |
| WT | Vehicle | 5 | 78±3 | 35±4 | 124±4 | 7.35±0.03 |
|  | TIMP1 (50 nM) | 5 | 79±2 | 37±3 | 126±5 | 7.35±0.03 |
| WT | Vehicle | 5 | 76±3 | 36±3 | 128±6 | 7.35±0.04 |
|  | TIMP2 (50 nM) | 5 | 76±5 | 35±2 | 122±5 | 7.34±0.03 |

All mice used in these studies are 2-month-old wild-type males. MAP, mean arterial pressure
