# Supplementary material for: Mechanistic insights into a TIMP3-sensitive pathway constitutively engaged in the regulation of cerebral hemodynamics
Source: eLife. 2016 Aug 1;5:e17536. doi: 10.7554/eLife.17536 (PMC4993587; doi:10.7554/eLife.17536)
Supplement: Figure 2—source data 1. — DOI: http://dx.doi.org/10.7554/eLife.17536.010 [file elife-17536-fig2-data1.docx]

## Figure 2- source data 1: Reagents used for Figure 2

| **Drug**  **(molecular weight, kDa)** | **Selectivity** | **Final concentration**  **(duration of superfusion)** |
| --- | --- | --- |
| **GW413333X** | ADAM10/ADAM17 inhibitor | 5µM  (30 min) |
| **GI254023X** | ADAM10 inhibitor | 5-20µM  (30 min) |
| **Soluble ectodomain**  **of human ADAM17**  **(64 kDa)** | ADAM17 substrates | 16 nM  (30 min) |
