# Supplementary material for: Mechanistic insights into a TIMP3-sensitive pathway constitutively engaged in the regulation of cerebral hemodynamics
Source: eLife. 2016 Aug 1;5:e17536. doi: 10.7554/eLife.17536 (PMC4993587; doi:10.7554/eLife.17536)
Supplement: Figure 2—source data 2. — DOI: http://dx.doi.org/10.7554/eLife.17536.011 [file elife-17536-fig2-data2.docx]

**Figure 2- source data 2: Main physiological variables of mice studied in Figure 2**

| Genotype  (age, m.o) | Treatment  (concentration) | N | MAP  (mmHg) | pCO_2_  (mmHg) | pO_2_  (mmHg) | pH |
| --- | --- | --- | --- | --- | --- | --- |
| *WT* (2) | Vehicle | 5 | 80±2 | 36±1 | 126±6 | 7.35±0.01 |
|  | GW (5 µM) | 5 | 81±3 | 36±1 | 126±4 | 7.36±0.01 |
| *WT* (2) | Vehicle | 5 | 81±2 | 36±1 | 124±3 | 7.34±0.01 |
|  | GI (5 µM) | 5 | 81±2 | 35±1 | 125±4 | 7.35±0.01 |
|  | GI (20 µM) | 5 | 82±2 | 35±2 | 127±5 | 7.34±0.02 |
| *Adam17^+/+^* (2) | Vehicle | 5 | 78±1 | 35±1 | 125±5 | 7.33±0.02 |
|  | sADAM17  (16 nM) | 5 | 78±1 | 36±1 | 123±3 | 7.34±0.02 |
| *Adam17^ex/+^* (2) | Vehicle | 5 | 74±1 | 35±2 | 122±3 | 7.33±0.02 |
|  | sADAM17  (16 nM) | 5 | 74±1 | 34±1 | 121±3 | 7.33±0.01 |
| *ADAM1 ^ex/ex^* (2) | Vehicle | 5 | 76±2 | 36±1 | 124±4 | 7.35±0.01 |
| *nonTg* (6) | Vehicle | 5 | 75±4 | 36±2 | 125±5 | 7.32±0.02 |
|  | sADAM17  (16 nM) | 5 | 78±4 | 34±3 | 125±6 | 7.33±0.03 |
| *TgBAC-TIMP3* (6) | Vehicle | 5 | 85±5*** | 37±2 | 129±3 | 7.33±0.04 |
|  | sADAM17  (16 nM) | 5 | 87±6*** | 36±3 | 130±5 | 7.34±0.02 |

All mice used in these studies are males. m.o., month-old; MAP, mean arterial pressure; **p*< 0.05 versus *nonTg*, one-way ANOVA followed by Tukey post hoc test
