# Supplementary material for: Mechanistic insights into a TIMP3-sensitive pathway constitutively engaged in the regulation of cerebral hemodynamics
Source: eLife. 2016 Aug 1;5:e17536. doi: 10.7554/eLife.17536 (PMC4993587; doi:10.7554/eLife.17536)
Supplement: Figure 3—source data 1. — DOI: http://dx.doi.org/10.7554/eLife.17536.016 [file elife-17536-fig3-data1.docx]

## Figure 3- source data 1: Reagents used for Figure 3

| **Drug**  **(molecular weight, kDa)** | **Selectivity** | **Final concentration**  **(duration of superfusion)** |
| --- | --- | --- |
| **Tyrphostin AG1478** | ErbB1 and ErbB4 inhibitor -  competitively binds to the ATP pocket of ErbB1 and ErbB4 | 10-20 µM  (30 min) |
| **Tyrphostin AG825** | ErbB2 inhibitor -  competitively binds to the ATP pocket of ErbB2 | 50-200 µM  (30 min) |
| **Murine ErbB1-human IgG1Fc chimera**  **(135-150 kDa)** | Blocks activation by EGF, TGFa, AREG or EPGN | 66.7 nM  (90 min) |
| **Human IgG1 Fc**  **(30-35 kDa)** | Control of ErbB1-human IgG1Fc | 286 nM  (90 min) |
| **Murine ErbB4-murine IgG2 Fc chimera**  **(120-140 kDa)** | Blocks activation by HB-EGF, EREG, BTC, NRG3 or NRG4 | 71.4 nM  (90 min) |
| **Murine ErbB3-murine IgG2 Fc chimera**  **(120-135 kDa)** | Blocks activation by  NRG1, NRG2 | 71.4 nM  (90 min) |
| **Murine IgG2a Fc**  **(35 kDa)** | Control of ErbB3 and ErbB4-human IgG2Fc | 286 nM  (90 min) |
| **Heparin** | competitively inhibits HB-EGF binding to its co-receptor heparan sulfate proteoglycan | 40 UI/ ml  (90 min) |
| **p21 peptide**  **(2.1 kDa)** | competitively inhibits HB-EGF binding to its co-receptor heparan sulfate proteoglycan | 12 µM  (90 min) |
| **p21^mut^ peptide**  **(2.1 kDa)** | Inactive version of the p21 peptide | 12 µM  (90 min) |
