# Supplementary material for: Mechanistic insights into a TIMP3-sensitive pathway constitutively engaged in the regulation of cerebral hemodynamics
Source: eLife. 2016 Aug 1;5:e17536. doi: 10.7554/eLife.17536 (PMC4993587; doi:10.7554/eLife.17536)
Supplement: Figure 3—source data 2. — DOI: http://dx.doi.org/10.7554/eLife.17536.017 [file elife-17536-fig3-data2.docx]

**Figure 3- source data 2: Main physiological variables of mice studied in Figure 3**

| Genotype | Treatment  (concentration) | N | MAP  (mmHg) | pCO_2_  (mmHg) | pO_2_  (mmHg) | pH |
| --- | --- | --- | --- | --- | --- | --- |
| WT | Vehicle | 5 | 79±4 | 35±2 | 126±4 | 7.33±0.02 |
|  | AG1478 (10 µM) | 5 | 80±3 | 35±1 | 126±7 | 7.34±0.02 |
|  | AG1478 (20 µM) | 5 | 80±2 | 35±1 | 125±4 | 7.36±0.02 |
| WT | Vehicle | 5 | 78±3 | 35±3 | 121±3 | 7.33±0.01 |
|  | AG825 (50 µM) | 5 | 79±3 | 35±1 | 122±4 | 7.34±0.03 |
|  | AG825 (200 µM) | 5 | 79±2 | 35±1 | 125±3 | 7.35±0.02 |
| WT | Vehicle | 5 | 81±1 | 35±2 | 123±4 | 7.35±0.01 |
|  | ErbB1- IgG1Fc  (66.7 nM) | 5 | 79±2 | 35±1 | 123±7 | 7.35±0.02 |
| WT | Vehicle | 5 | 79±2 | 35±1 | 124±5 | 7.34±0.02 |
|  | IgG1 Fc (286 nM) | 5 | 80±2 | 35±1 | 125±4 | 7.34±0.02 |
| WT | Vehicle | 5 | 80±2 | 36±1 | 125±5 | 7.33±0.02 |
|  | ErbB4- IgG2Fc (71.4 nM) | 5 | 79±2 | 35±1 | 124±3 | 7.34±0.02 |
| WT | Vehicle | 5 | 81±2 | 35±1 | 126±5 | 7.34±0.01 |
|  | IgG2Fc (286 nM) | 5 | 80±2 | 36±1 | 125±4 | 7.34±0.02 |
| WT | Vehicle | 5 | 80±2 | 35±1 | 121±5 | 7.34±0.01 |
|  | ErbB3- IgG2Fc (71.4 nM) | 5 | 80±3 | 35±2 | 123±3 | 7.34±0.01 |
| WT | IgG1Fc+IgG2Fc  (286 nM each) | 5 | 80±2 | 36±2 | 124±7 | 7.35±0.03 |
|  | ErrB1- IgG1Fc + ErbB4- IgG2Fc  (66.7nM-71.4nM) | 5 | 79±3 | 35±3 | 124±5 | 7.34±0.03 |
| WT | Vehicle | 5 | 82±2 | 36±1 | 126±5 | 7.34±0.01 |
|  | Heparin  (40U/mL) | 5 | 80±1 | 35±1 | 127±5 | 7.34±0.03 |
| WT | Vehicle | 5 | 81±2 | 35±1 | 126±5 | 7.35±0.02 |
|  | p21 peptide  (12 µM) | 5 | 81±2 | 36±2 | 126±5 | 7.34±0.02 |
| WT | Vehicle | 5 | 81±2 | 36±1 | 125±4 | 7.33±0.02 |
|  | p21-mut peptide (12 µM) | 5 | 79±2 | 35±2 | 127±4 | 7.34±0.02 |

All mice used in these studies are 2-month-old wild-type males. MAP, mean arterial pressure
