# Supplementary material for: Mechanistic insights into a TIMP3-sensitive pathway constitutively engaged in the regulation of cerebral hemodynamics
Source: eLife. 2016 Aug 1;5:e17536. doi: 10.7554/eLife.17536 (PMC4993587; doi:10.7554/eLife.17536)
Supplement: Figure 4—source data 1. — DOI: http://dx.doi.org/10.7554/eLife.17536.021 [file elife-17536-fig4-data1.docx]

## Figure 4- source data 1: Reagents used for Figure 4

| **Drug**  **(molecular weight, kDa)** | **Selectivity** | **Final concentration**  **(duration of superfusion)** |
| --- | --- | --- |
| **Tyrphostin AG1478** | ErbB1 and ErbB4 inhibitor -  competitively binds to the ATP pocket of ErbB1 and ErbB4 | 10-20 µM  (30 min) |
| **Soluble ectodomain of HB-EGF**  **(10 kDa)** | ErbB1 and ErbB4 | 20 nM  (30 min) |
| **Murine TIMP3**  **(24-28 kDa)** | Inhibits all MMPs, ADAM10 and ADAM17, ADAMTS | 40 nM  (30 min) |
| **GW413333X** | ADAM10/ADAM17 inhibitor | 5µM  (30 min) |
