# Supplementary material for: Mechanistic insights into a TIMP3-sensitive pathway constitutively engaged in the regulation of cerebral hemodynamics
Source: eLife. 2016 Aug 1;5:e17536. doi: 10.7554/eLife.17536 (PMC4993587; doi:10.7554/eLife.17536)
Supplement: Figure 4—source data 2. — DOI: http://dx.doi.org/10.7554/eLife.17536.022 [file elife-17536-fig4-data2.docx]

**Figure 4- source data 2: Main physiological variables of mice studied in Figure 4**

| Genotype | Treatment  (concentration) | N | MAP  (mmHg) | pCO_2_  (mmHg) | pO_2_  (mmHg) | pH |
| --- | --- | --- | --- | --- | --- | --- |
| WT | Vehicle | 5 | 81±2 | 36±1 | 127±2 | 7.34±0.01 |
|  | sHB-EGF (20 nM) | 5 | 81±2 | 36±1 | 125±4 | 7.35±0.01 |
|  | sHB-EGF (20 nM) +  AG1478 (20µM) | 5 | 80±2 | 35±1 | 124±4 | 7.35±0.01 |
| WT | Vehicle | 5 | 80±2 | 35±2 | 125±3 | 7.35±0.01 |
|  | TIMP3 (40 nM) | 5 | 80±3 | 35±2 | 125±3 | 7.35±0.02 |
|  | TIMP3 (40 nM) +  sHB-EGF (20 nM) | 5 | 80±3 | 35±2 | 125±3 | 7.35±0.02 |
| WT | Vehicle | 5 | 80±2 | 36±1 | 126±6 | 7.35±0.01 |
|  | GW (5 µM) | 5 | 81±3 | 36±1 | 126±4 | 7.36±0.01 |
|  | GW (5 µM) +  sHB-EGF (20 nM) | 5 | 80±2 | 36±1 | 128±5 | 7.35±0.02 |

All mice used in these studies are 2-month-old wild-type males. MAP, mean arterial pressure
