# Supplementary material for: Mechanistic insights into a TIMP3-sensitive pathway constitutively engaged in the regulation of cerebral hemodynamics
Source: eLife. 2016 Aug 1;5:e17536. doi: 10.7554/eLife.17536 (PMC4993587; doi:10.7554/eLife.17536)
Supplement: Figure 5—source data 1. — DOI: http://dx.doi.org/10.7554/eLife.17536.027 [file elife-17536-fig5-data1.docx]

## Figure 5- source data 1: Reagents used for Figure 5

| **Drug**  **(molecular weight, kDa)** | **Selectivity** | **Final concentration**  **(duration of incubation)** |
| --- | --- | --- |
| **Tyrphostin AG1478** | ErbB1 and ErbB4 inhibitor -  competitively binds to the ATP pocket of ErbB1 and ErbB4 | 2 µM  (20 min) |
| **GW413333X** | ADAM10/ADAM17 inhibitor | 1µM  (20 min) |
| **GI254023X** | ADAM10 inhibitor | 1µM  (20 min) |
| **Murine TIMP2**  **(23 kDa)** | Inhibits all MMPs, ADAM12 | 10 nM  (20 min) |
| **Murine TIMP3**  **(24-28 kDa)** | Inhibits all MMPs, ADAM10 and ADAM17, ADAMTS | 4-8 nM  (20 min) |
| **Heparin** | competitively inhibits HB-EGF binding to its co-receptor heparan sulfate proteoglycan | - |
| **Soluble ectodomain of HB-EGF**  **(10 kDa)** | ErbB1 and ErbB4 | 3 nM  (20 min) |
| **p21 peptide**  **(2.1 kDa)** | competitively inhibits HB-EGF binding to its co-receptor heparan sulfate proteoglycan | 2.4 µM  (60 min) |
| **p21^mut^ peptide**  **(2.1 kDa)** | Inactive version of the p21 peptide | 2.4 µM  (60 min) |
