# Supplementary material for: Mechanistic insights into a TIMP3-sensitive pathway constitutively engaged in the regulation of cerebral hemodynamics
Source: eLife. 2016 Aug 1;5:e17536. doi: 10.7554/eLife.17536 (PMC4993587; doi:10.7554/eLife.17536)
Supplement: Figure 6—source data 1. — DOI: http://dx.doi.org/10.7554/eLife.17536.032 [file elife-17536-fig6-data1.docx]

**Figure 6- source data 1:**  **Main physiological variables of mice studied in Figure 6**

| Genotype  (age, m.o) | Treatment  (concentration) | N | MAP  (mmHg) | pCO_2_  (mmHg) | pO_2_  (mmHg) | pH |
| --- | --- | --- | --- | --- | --- | --- |
| *Non-Tg* (6) | Vehicle | 6 | 75±4 | 36±2 | 125±5 | 7.32±0.01 |
|  | sADAM17 (16 nM) | 6 | 78±4 | 34±3 | 125±6 | 7.34±0.03 |
| *TgNotch3^R169C^* (6) | Vehicle | 5 | 78±4 | 36±3 | 132±7 | 7.34±0.04 |
|  | sADAM17 (16 nM) | 5 | 75±6 | 35±3 | 124±2 | 7.36±0.03 |
| *Non-Tg* (6) | Vehicle | 5 | 81±2 | 36±1 | 127±2 | 7.34±0.01 |
|  | sHB-EGF (20 nM) | 5 | 81±2 | 35±2 | 125±5 | 7.35±0.01 |
| *TgNotch3^R169C^* (6) | Vehicle | 5 | 80±2 | 35±1 | 127±4 | 7.35±0.02 |
|  | sHB-EGF (20 nM) | 5 | 80±3 | 35±2 | 124±5 | 7.35±0.02 |

All mice used in these studies are males. m.o., month-old MAP, mean arterial pressure
