# Supplementary material for: Mechanistic insights into a TIMP3-sensitive pathway constitutively engaged in the regulation of cerebral hemodynamics
Source: eLife. 2016 Aug 1;5:e17536. doi: 10.7554/eLife.17536 (PMC4993587; doi:10.7554/eLife.17536)
Supplement: Figure 7—source data 1. — DOI: http://dx.doi.org/10.7554/eLife.17536.036 [file elife-17536-fig7-data1.docx]

**Figure 7- source data 1:**  **Comparison of cerebral K_V_ current properties.**

| **Cerebral myocytes** | **V _½_ (mV)** | **Slope k (mV)** | **τ_act_ (-80 to +40mV, ms)** | **τ_deact_ (+40 to -40mV, ms)** |
| --- | --- | --- | --- | --- |
| *TgNotch3^R169C^; Timp3^+/+^* | 3.80 ± 0.80 | 16.97 ±0.81 | 38.96 ±3.08 | 28.03 ±2.68 |
| *TgNotch3^R169C^; Timp3^+/-^* | 3.38 ±0.97 | 14.09 ±0.93 | 36.84 ±5.47 | 28.07 ±2.36 |
| *TgNotch3^R169^*^C^/sADAM17 | 7.63 ±1.15 | 16.46 ±1.12 | 36.86 ±3.36 | 27.76 ±3.11 |
| *WT* | 6.28 ±0.92 | 13.92 ±0.87 | 41.38 ±2.42 | 32.85 ±3.08 |
| *WT*/TIMP3 | 4.28 ±1.56 | 15.13 ±1.52 | 42.08 ±2.46 | 34.17 ±4.19 |

Half-maximal activation (V_1/2_) voltage, slope (k), activation time constant (τ_act_), and deactivation time constant (τ_deac_) were used as channel fingerprints to compare K_V_ current properties in the different conductions. The similarity in the measured parameters points to a modulation of the number of channel rather than changes in gating properties or subtypes expression. Comparison of kinetic and activation properties suggests a predominant role of K_v_ 1.5 subtype in the recorded currents (Dabertrand et al., 2015).
